# Implications of miRNAs dysregulation in amyotrophic lateral sclerosis: Challenging for clinical applications

**DOI:** 10.3389/fnins.2023.1131758

**Published:** 2023-02-21

**Authors:** Yuka Koike, Osamu Onodera

**Affiliations:** Department of Neurology, Brain Research Institute, Niigata University, Niigata, Japan

**Keywords:** amyotrophic lateral sclerosis, micro RNAs, regulators of gene expression, TDP-43, FUS, SOD1, biomarkers

## Abstract

Amyotrophic lateral sclerosis (ALS) is a fatal neurodegenerative disease characterized by the selective degeneration of upper and lower motor neurons. Currently, there are no effective biomarkers and fundamental therapies for this disease. Dysregulation in RNA metabolism plays a critical role in the pathogenesis of ALS. With the contribution of Next Generation Sequencing, the functions of non-coding RNAs (ncRNAs) have gained increasing interests. Especially, micro RNAs (miRNAs), which are tissue-specific small ncRNAs of about 18–25 nucleotides, have emerged as key regulators of gene expression to target multiple molecules and pathways in the central nervous system (CNS). Despite intensive recent research in this field, the crucial links between ALS pathogenesis and miRNAs remain unclear. Many studies have revealed that ALS-related RNA binding proteins (RBPs), such as TAR DNA-binding protein 43 (TDP-43) and fused in sarcoma/translocated in liposarcoma (FUS), regulate miRNAs processing in both the nucleus and cytoplasm. Of interest, Cu^2+^/Zn^2+^ superoxide dismutase (SOD1), a non-RBP associated with familial ALS, shows partially similar properties to these RBPs *via* the dysregulation of miRNAs in the cellular pathway related to ALS. The identification and validation of miRNAs are important to understand the physiological gene regulation in the CNS, and the pathological implications in ALS, leading to a new avenue for early diagnosis and gene therapies. Here, we offer a recent overview regarding the mechanism underlying the functions of multiple miRNAs across TDP-43, FUS, and SOD1 with the context of cell biology, and challenging for clinical applications in ALS.

## 1. Introduction

Amyotrophic lateral sclerosis (ALS) is a late-onset neurodegenerative disorder characterized by the progressive degeneration of motor neurons in the motor cortex, brainstem and spinal cord. Its main clinical features are paralysis of voluntary muscles, muscle atrophy and fasciculation (Hardiman et al., 2017). It usually results in death within 3–5 years from the onset due to respiratory dysfunction (Amado and Davidson, 2021). ALS includes sporadic ALS (sALS) and familial ALS (fALS). Only 5–10% of ALS are familial cases. However, 10% of initially diagnosed sALS patients have gene mutations (Chio et al., 2013). The mutant genes include Cu^2+^/Zn^2+^ superoxide dismutase (*SOD1*), TAR DNA-binding protein 43 (*TARDBP*), fused in sarcoma/translocated in liposarcoma (*FUS/TLS*), hexanucleotide expansion repeat in chromosome 9 open reading frame 73 (*C9orf72*), Optineurin (*OPTN*), TANK-binding kinase 1(*TBK1*) and Matrin 3 (MATR3) (Brenner and Weishaupt, 2019). Of importance, many ALS-linked genes, such as *TARDBP* and *FUS*, deeply contribute to RNA metabolism, including molecular processes of non-coding RNAs (ncRNAs) (Lagier-Tourenne et al., 2010; Kawahara and Mieda-Sato, 2012).

The Human Genome Project revealed that in the three billion bases of the human genome, only ~2% encode protein, which means that the most portion of genome produces many so-called “ncRNAs” (Djebali et al., 2012) NcRNAs are very diversified including small nucleolar RNAs, small interfering RNAs, microRNAs (miRNAs), circular RNAs and long-non-coding RNAs. The common theme for all of these ncRNAs is their function contributing to gene expression, which is involved in extensive cellular metabolism such as development, differentiation, cell death, transcriptional process and post-transcriptional modifications (Ma et al., 2020). MiRNAs are evolutionally conserved, tissue-specific small ncRNAs of about 18–25 nucleotides, with a role as important post-transcriptional regulators of gene expression (Krol et al., 2010; Bartel, 2018). MiRNAs repress gene expression by binding to complementary sequences in the 3'UTRs of target messenger RNA (mRNA), which leads to inhibition of protein synthesis by inducing mRNA destabilization or repressing mRNA translation (Huntzinger and Izaurralde, 2011). While a single miRNA controls multiple different genes, the expression of a single gene is regulated by a network of many interactive miRNAs (Lim et al., 2005; Guo et al., 2010; Peter, 2010; Wu et al., 2010).

Recent clarifications of the detailed roles of miRNAs have provided new insights into the regulation of gene expression in neurodegenerative diseases, including various forms of ALS (Rinchetti et al., 2018; Laneve et al., 2021). It also indicates that miRNAs could be effective biomarkers in neurodegenerative diseases (Ruffo et al., 2021; Liu et al., 2022). Here, we summarize (1) the mechanisms by which miRNAs contribute to ALS pathogenesis, and (2) their potential as biomarkers and therapeutic targets for ALS.

## 2. The role of miRNAs in ALS pathogenesis

Most of the human canonical miRNAs are encoded by intronic region of the functional genes. The biogenesis of miRNAs is regulated by two RNase III enzymes, Drosha and Dicer, which catalyze in the nucleus and cytoplasm, respectively (Michlewski and Caceres, 2019). This biogenesis process starts in the nucleus with transcription of a long primary miRNA (pri-mRNA) from genome DNA by RNA polymerase II. Then, pri-mRNA is cleaved by Drosha to form a precursor miRNA (pre-miRNA) and transported to the cytoplasm by Exportin-5, nuclear export receptor. In the cytoplasm, Dicer binds pre-miRNA and dissects into miRNA duplex (double-strand miRNA). The guide strand of the duplex (mature single-strand miRNA) is incorporated into the RNA-induced silencing complex (RISC), which binds to the 3'UTR of the target mRNA for gene regulation, whereas the other strand is usually degraded (Cassidy et al., 2019; Chen et al., 2019; Pu et al., 2019; Saliminejad et al., 2019).

**Figure 1:**
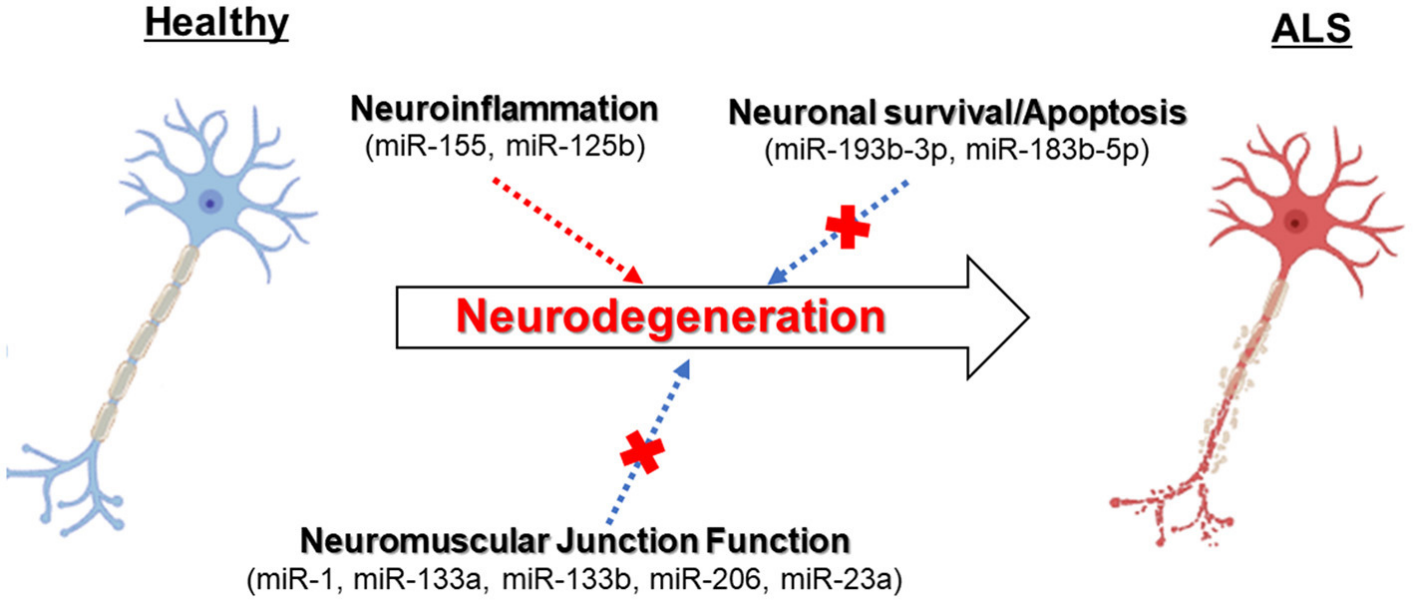
The dysregulation of miRNAs can contribute to neurodegeneration in ALS motor neurons related to several molecular pathways.

Multiple studies have revealed that biogenetic factors of miRNAs are affected in ALS (Campos-Melo et al., 2013; Emde et al., 2015; Figueroa-Romero et al., 2016) (Figure 1). Overall, the reduced levels of specific subsets of miRNAs could be detected in fALS and sALS compared to healthy individuals and patients with other neurodegenerative diseases (Campos-Melo et al., 2013; Figueroa-Romero et al., 2016). Elucidating the mechanisms by which miRNA dysfunction is involved in ALS leads to developing disease biomarkers and therapeutic strategies. Here, we subdivide and describe the four major roles implicated in ALS pathogenesis.

### The biosynthesis of miRNAs and ALS-related RNA-binding proteins

It has been highlighted the connections between miRNAs and ALS-related RNA-binding proteins (RBPs), TDP-43 and FUS, with essential regulatory complexes such as Drosha and in the nucleus and Dicer in the cytoplasm (Ling et al., 2010). TDP-43 and FUS are ubiquitously expressed RBPs regulating many aspects of RNA metabolism and processing. Nuclear depletion and cytoplasmic inclusion of TDP-43 is a pathological hallmark in more than 97% of ALS cases (Lagier-Tourenne et al., 2010; Taylor et al., 2016) whereas FUS inclusions are less common (Ling et al., 2013). Nuclear TDP-43 binds to the selected pri-miRNAs and promotes the cleavage of them by Drosha. Cytoplasmic TDP-43 binds to the terminal loops of the pre-miRNAs and promotes the cleavage of the specific pre-miRNAs by Dicer (Kawahara and Mieda-Sato, 2012). FUS binds the specific pri-miRNAs and cooperates with co-transcriptional Drosha recruitment on chromatin sites, which enhance the synthesis of the specific miRNAs (Morlando et al., 2012). Indeed, it has been shown that the expressions of the subsets of miRNAs are altered by knockdown of *TARDBP* or *FUS/TLS* (Kawahara and Mieda-Sato, 2012; Morlando et al., 2012). Furthermore, ALS-linked TDP-43 mutants showed the different binding patterns of mature miRNAs from wild-type and sequestered miRNAs in cytoplasmic inclusion (Paez-Colasante et al., 2020; Zuo et al., 2021). These findings indicate the link between altered miRNAs biogenesis and impaired Drosha and Dicer processing mechanism, as possible pathological implications in ALS.

### MiRNAs and the dysfunction of neuromuscular junctions

Many studies have revealed that ALS is a multi-systemic disease, beyond the motor neuron system, including glial cells and muscle cells, are involved in the pathogenesis. Recently, it has been shaded light on the mechanisms underlying microRNAs dysregulation in the skeletal muscles involved in ALS pathogenesis. MiRNAs specifically expressed in the skeletal muscles have been identified as myomiRs such as miR-1, miR-133a, miR-133b, miR-206, and miR-23a, which take an important part in the molecular network controlling myogenesis *via* myogenic transcriptional factors (Di Pietro et al., 2018). Especially, miR-206, expressed in the skeletal muscles with physiological conditions, is involved in the maintenance of neuromuscular junctions and synapses, regulating myoblast differentiation (Toivonen et al., 2014). In addition, Williams et al. (2009) found that miR-206 suppresses the expression levels of muscular histone deacethylase 4 (HDAC4), which is an inhibitor of neuromuscular junction re-innervation *via* fibroblast growth factor binding protein 1 (FGFBP1), indicating the effect of miR-206 on promoting re-innervation after injury. Moreover, King et al. (2014) showed that TDP-43 associates with the mature miR-1/miR-206 family in muscle cells.

Based on these findings, Pegoraro et al. assessed the expression levels of multiple myomiRs in skeletal muscular biopsies derived from patients with sALS and fALS, including *SOD1* and *C9orf72* mutation carriers. They revealed that miR-1, miR-133a, and miR-133b were downregulated in muscle specimens from sALS compared to those from healthy individuals. On the other hand, miR-206 was upregulated in the muscles from patients with *SOD1* and *C9orf72* mutations (Pegoraro et al., 2020). Related to these myomiRs, Nie et al. (2015) showed miR-1 is required for muscle differentiation through acting on HDAC6, while miR-133 induces muscle proliferation.

Furthermore, Russell et al. (2013) showed that not only miR-206, miR-23a, miR-29b, miR-31, and miR-455 were upregulated in ALS patients compared with healthy individuals. Among these miRNAs, only miR-23a, which was also dysregulated in SOD1^G93A^ mice (Williams et al., 2009), was more significantly increased in the ALS than in neurological disease controls. They demonstrated that miR-23a suppresses the expression levels of peroxisome proliferator-activated receptor gamma coactivator 1-alpha (PGC-1α), which is involved in mitochondrial biogenesis and function (Russell et al., 2013). These studies indicate that multiple myomiRs can contribute to ALS pathogenesis *via* functionally different roles during muscle development and re-innervation processes.

### MiRNAs and neuroinflammation

In ALS, neuroinflammation takes a significant role in the disease progression *via* immune system such as microglial activation and dysregulation of immune-related genes (Rinchetti et al., 2018). One of the well-studied miRNAs is the inflammatory miR-155, which is upregulated in fALS and sALS patients, and pre-symptomatic SOD1^G93A^ mice (Koval et al., 2013; Butovsky et al., 2015; Cunha et al., 2018). Of particular interest, Koval et al. (2013) demonstrated that the miR-155 levels in cerebrospinal fluid from ALS patients are twice as elevated as those from control individuals. Furthermore, miR-155 is involved in the elevation of proinflammatory cytokine secretion by binding to a repressor of cytokine signaling 1 (SOCS1) mRNAs (O'Connell et al., 2007, 2010; Ceppi et al., 2009). Indeed, the anti-miR-155 could significantly prolong the survival time in SOD1^G93A^ mice (Koval et al., 2013).

Moreover, Parisi et al. (2013, 2016) investigated the modulating role of miR-125b in the maintenance of NF-kb signals in microglia. They demonstrated that miR-125b activates NF-kb in microglia with toxic effects on surrounding motor neurons and the inhibition of this miRNA reduces the expression of the transcriptional targets of NF-kb, protecting motor neurons in SOD1^G93A^ mice model (Parisi et al., 2016). These results enhance the important role of inflammatory miRNAs in the connection between microglia and motor neurons, which is attracting attention as a contributor to motor neuron diseases.

### MiRNAs and dysfunction of neuronal survival/apoptosis

Li et al. showed that miR-193b-3p is downregulated in SOD1^G93A^ mice as well as in patients with ALS. They showed that miR-193b-3p, which targets tuberous sclerosis 1 (TSC1) and regulates the activity of mechanistic target of rapamycin complex 1 (mTORC1), induces cell death using mouse motor neuron-like cells (NSC-34 cells). Since miR-193b-3p downregulates autophagy mechanism, reducing this miRNA is required for cell survival by promoting autophagy through TSC1-mTORC1 pathway (Li et al., 2017). Additionally, they found that miR-183b-5p regulates apoptosis and necrosis pathways by targeting RIP kinase 1 (RIPK) and programmed cell death 4 (PDCD4), suggesting the role of miR-183b-5p as a mediator of programmed neuronal death (Li et al., 2020). These studies indicate the link between regulating miRNAs and cell survival as a common mechanism in ALS regardless of gene mutations, which provides a future perspective for therapeutic strategy.

## 3. MiRNAs as potential biomarkers in ALS

A huge number of studies focused on elucidating the role of miRNAs as potential biomarkers for accurate diagnosis, prediction of the prognosis and monitoring the progression of ALS. Based on high-throughput methods including the use of Next Generation Sequencing, the signatures of miRNAs were identified in the easy-to-reach biological specimens, such as circulating body fluids and muscle biopsies, from patients with ALS (Goodall et al., 2013; Di Pietro et al., 2018; Foggin et al., 2019; Joilin et al., 2019; Juzwik et al., 2019; Ravnik-Glavac and Glavac, 2020; Laneve et al., 2021; Liu et al., 2022). Thus, we summarize recent attempts and limitations in finding effective biomarkers targeted at the alterations or dysfunction of miRNAs in human ALS biological samples (Table 1).

**Table 1:** Altered miRNAs in samples from ALS patients.

**miRNAs**	**Target tissues**	**Direction of regulation in ALS patients**	**References**
miR-206	Muscle	Up	Russell et al. (2013), Pegoraro et al. (2020)
miR-23a	Muscle	Up	Russell et al. (2013)
miR-29b	Muscle	Up	Russell et al. (2013)
miR-31	Muscle	Up	Russell et al. (2013)
miR-455	Muscle	Up	Russell et al. (2013)
miR-1	Muscle/serum	Down (muscle)/up (serum)	Raheja et al. (2018), Pegoraro et al. (2020)
miR-133a	Muscle/serum	Down (muscle)/up (serum)	Raheja et al. (2018), Pegoraro et al. (2020)
miR-133b	Muscle/serum	Down (muscle)/up (serum)	Raheja et al. (2018), Pegoraro et al. (2020)
miR19a-3p	Serum	Up	Raheja et al. (2018)
miR-144-5p	Serum	Up	Raheja et al. (2018)
miR-192-5p/3p	Serum	Up	Raheja et al. (2018)
miR-139-5p	Serum	Down	Raheja et al. (2018)
miR-320a	Serum	Down	Raheja et al. (2018)
miR-320b	Serum	Down	Raheja et al. (2018)
miR-320c	Serum	Down	Raheja et al. (2018)
miR-425-5p	Serum	Down	Raheja et al. (2018)
let-7d-3p	Serum	Down	Raheja et al. (2018)
miR-1825	Serum	Down	Freischmidt et al. (2015)
miR-1234-3p	Serum	Down	Freischmidt et al. (2015)
miR-181	Plasma	Up	Magen et al. (2021)
miR-193b-3p	Leucocyte	Down	Chen et al. (2019)
miR-155	CSF	Up	Koval et al. (2013)
miR-132-5p/3p	CSF	Down	Freischmidt et al. (2013)
miR-574-5p/3p	CSF	Down	Freischmidt et al. (2013)
miR-124-3p	CSF	Down	Yelick et al. (2020)

### Serum/plasma miRNAs in ALS

Several studies focused on the serum and plasma from patients with sALS and fALS, and demonstrated the alteration of miRNAs expression profiles. Freishmidt et al. reported that 24 miRNAs were downregulated in serum from fALS patients and/or asymptomatic ALS mutation carriers. Of interest, GDCGG and SGGC motifs were independently identified as highly enriched in these downregulated miRNAs (Freischmidt et al., 2014). The same authors also revealed that miR-1825 and miR-1234-3p were downregulated in serum from sALS patients (Freischmidt et al., 2015). These studies showed that homogenous miRNAs, which have consistent sequence motifs, are downregulated in ALS caused by different genes. Reheja et al. revealed that seven miRNAs: miR-1, miR-19a-3p, miR-133b, miR-133a-3p, miR-144-5p, miR-192-3p, and miR-192-5p were upregulated and six miRNAs: miR-139-5p, miR-320a, miR-320b, miR-320c, miR-425-5p, and let-7d-3p were downregulated in ALS patients in comparison with healthy controls and neurological disease controls such as Alzheimer's disease and multiple sclerosis. They also demonstrated that the alteration of these miRNAs levels was associated with clinical parameters over time, suggesting that they could act as prognostic markers (Raheja et al., 2018).

Magen et al. evaluated the plasma miRNAs as candidates for prognostic biomarkers in 252 patients with ALS. They demonstrated that the expression levels of miR-181, which is enriched in neurons and hematopoietic tissues, are stable during the course of disease. Then, the authors showed that high levels of miR-181 predict shorter survival time compared to low levels of miR-181 (low miR-181, median 38.9-month, high miR-181, median: 28.8-month, log-rank χ^2^ = 16.7, *p* = 0.0001), suggesting that miR-181 functions as a potential prognostic biomarker for ALS. Moreover, they revealed that the evaluation of miR-181 can enhance the prognostic value of neurofilament light chain (NfL), a protein biomarker already known in ALS. The authors stratified samples from 243 patients into tertiles according to NfL levels. Importantly, higher miR-181 levels predicted shorter survival duration in the intermediate NfL level (HR = 2.37, 95% CI: 1.4–4.02, *p* = 0.001). Within the intermediate NfL tertile, lower miR-181 group had median 18-month longer survival duration than higher miR-181 group (log-rank χ^2^ = 51.1, *p* < 0.0001), it could not be detected by NfL alone assessment (Magen et al., 2021). Thus, the joint of miRNA–protein biomarker approach provides a more accurate prognostication compared to measuring individual one and enhances the potential significance of miRNAs as adjuvant diagnostic tools.

### Cerebrospinal fluid miRNAs in ALS

As attention has been focused on the cerebrospinal fluid (CSF) as the source that most reflects the neuropathophysiological status of CNS, studies targeting miRNAs in CSF have been attempted (Freischmidt et al., 2013; Benigni et al., 2016; Waller et al., 2017; Yelick et al., 2020). Freischmidt et al. evaluated whether the TDP-43 binding microRNAs identified in cell lines are dysregulated in sALS patients. They revealed that downregulation of miR-132-5p/3p and miR-574-5p/3p was apparent in ALS patients, including those with *TARDBP, FUS*, and *C9orf72* mutations, but not in those with *SOD1* mutation (Freischmidt et al., 2013). Yelick et al. demonstrated the downregulation of miR-124-3p with spinal motor neuron-derived exosomes in SOD1^G93A^ mice, including the pre-symptomatic stage. Also, in their study, miR-124-3p levels in CSF exosomes from sALS patients showed a significant positive correlation between CSF exosomal miR-124-3p levels and clinical functional score in ALS (ALSFRS-R) (Yelick et al., 2020). These miRNAs' diagnostic and prognostic utility requires further validation.

### Limitations of miRNAs as biomarkers

There are still two major problems with the use of miRNA-based biomarkers as diagnostic tools for ALS. First, it is unclear whether the altered levels of miRNAs detected in patients truly reflect neurodegeneration due to ALS. The majority of patients with sporadic ALS are elderly and have other diseases, which may affect the composition of their miRNAs. Second, miRNAs are unstable and easily degraded, causing the difficulty treating as biomarkers than proteins. In particular, it is known that the conditions of centrifugation and/or temperature after sample collection can much affect the quality of RNAs (McDonald et al., 2011; Kirschner et al., 2013). Currently, miRNA-based biomarkers are not superior to other biomarkers. In order to make the most effective and accurate use of miRNAs in the diagnostic field, as the first step, we may need to seek a way to combine with other established biomarkers, such as NfL.

## 4. Therapeutic strategies by miRNAs for ALS

Many researchers have been elucidating the roles of miRNAs in ALS pathology and have detected dysregulation of miRNAs in animal models, postmortem brains and biological specimens in ALS, leading to the potential of miRNAs as novel therapeutic targets for ALS. There are two major therapeutic strategies by miRNAs. One is anti-miRNA oligonucleotides (AMOs) therapy, and the other is miRNAs replacement therapy. When the upregulation of miRNAs associates with ALS pathogenesis, the expression of the target miRNAs can be inhibited by the binding of AMOs. On the other hand, when the deficient of miRNAs contributes to the pathogenesis, the deficient target miRNAs can be supplied by miRNAs mimics containing the same sequence as mature endogenous miRNAs (Bai et al., 2019; Liu et al., 2022). Moreover, recent advances in gene-modulating technology and delivery methods, including viral-based and non-viral based, have made miRNAs feasible therapeutic tools (Yang, 2015; Gaj et al., 2017; Lim et al., 2020).

While miRNA-based strategies are promising, there is still a long way to move toward their clinical application. Although several clinical trials are ongoing to test miRNA-based therapeutics against peripheral diseases like hepatitis C (Lanford et al., 2010), no therapeutics have reached clinical trials in neurodegenerative disease. Even in such a situation, Gemfibrozil, a drug to decrease cholesterol, has undergone a phase I trial to evaluate its ability to increase miR-107 levels for preventing Alzheimer's disease (NCT02045056). In this trial, 48 control and 24 mild cognitive impairment individuals were treated with gemfibrozil or placebo. As a result, gemfibrozil appeared to increase in miR-107 plasma levels and show trends for decrease of brain atrophy in the treatment group. However no significant differences have been observed (Walgrave et al., 2021). As a critical issue, such studies cannot directly access the efficacy of miRNA-based oligonucleotide therapy. Breakthroughs that bridge the gap between evolving miRNA research including gene manipulation technology, and clinical applications are highly desirable.

## 5. Discussion

Despite enormous efforts, many aspects of pathogenesis of ALS remain unknown, and there are no effective therapies for the disease. Elucidating previously unrecognized molecular mechanisms in ALS, such as miRNAs, could open up new chapters. As described here, the roles of miRNAs as key gene regulators of several significant cellular pathways support their involvement in ALS. Both of the dysregulation in proteins critical in miRNAs biogenesis (such as TDP-43, FUS), and the altered expression of the genes crucial for motor neuronal functions by miRNAs could play essential pathogenetic roles in ALS.

One of the reasons for the difficulty in understanding the disease is its phenotypically and genetically heterogeneity (Brenner and Weishaupt, 2019). The ALS-causative proteins, TDP-43 and FUS are important RBPs which regulate RNA metabolism. Especially, TDP-43 pathology in motor neurons is observed in the vast majority of ALS cases (Taylor et al., 2016). While SOD1 affects the stability and function of RNAs, SOD1 mutation animal models and patients do not display TDP-43 pathological changes (Polymenidou et al., 2012). Nevertheless, previous studies related to biological roles of miRNAs and biomarkers have found alterations in many miRNAs that are common in sALS patients and SOD1 transgenic mice. This suggests that loss or gain of functions of common miRNAs, regardless of the causative proteins, contributes to the onset and progression of the disease *via* the critical cellular pathways. However, more detailed validation of ALS models other than SOD1 transgenic mice is required to prove it.

Recent advances in research on patient-derived biofluids have demonstrated the potential of many miRNAs as biomarkers, especially combined with established protein biomarkers. In this context, it is important to individually verify whether they have values for the diagnosis in the early disease stages or preclinical phase, the appropriate prognostic prediction, and the evaluation of therapeutic efficacy. This field of research needs further investigation for clinically meaningful applications.

## Author contributions

YK wrote the manuscript with substantial support from OO. All authors agree to be accountable for the content of the work.
